# Supplementary material for: Single-dispersed polyoxometalate clusters embedded on multilayer graphene as a bifunctional electrocatalyst for efficient Li-S batteries
Source: Nat Commun. 2022 Jan 11;13:202. doi: 10.1038/s41467-021-27866-5 (PMC8752791; doi:10.1038/s41467-021-27866-5)
Supplement: Supplementary file 1 — Supplementary Information [file 41467_2021_27866_MOESM1_ESM.pdf]

SUPPLEMENTARY INFORMATION

Single-dispersed Polyoxometalate Clusters Embedded on Multilayer Graphene as a Bifunctional Electrocatalyst for Efficient Li-S Batteries

Jie Lei[1], Xiao-Xiang Fan[1], Ting Liu[1], Pan Xu[1], Qing Hou[1], Ke Li[1], Ru-Ming Yuan[1], Ming-Sen Zheng[1], Quan-Feng Dong[1*] and Jia-Jia Chen[1*]

[1] State Key Laboratory for Physical Chemistry of Solid Surfaces, Department of Chemistry, College of Chemistry and Chemical Engineering, iChem (Collaborative Innovation Center of Chemistry for Energy Materials), Innovation Laboratory for Sciences and Technologies of Energy Materials of Fujian Province (IKKEM), Xiamen University, Xiamen, Fujian, 361005, China.

*Corresponding author e-mail: *qfdong@xmu.edu.cn; JiaJia.Chen@xmu.edu.cn*

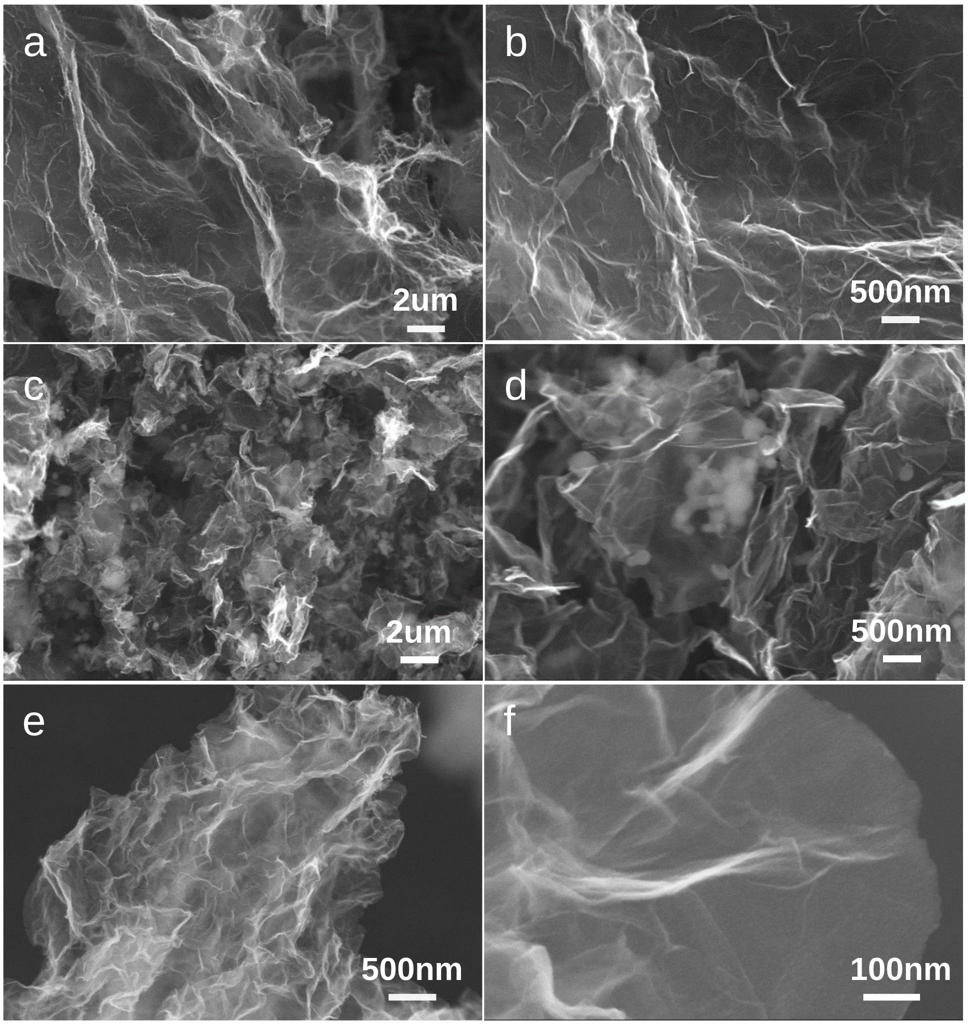

Supplementary Figure 1 SEM images of materials: (a-b) rGO, (c-d) {Co_4W_{18}}+rGO, and (e-f) {Co_4W_{18}}/rGO.

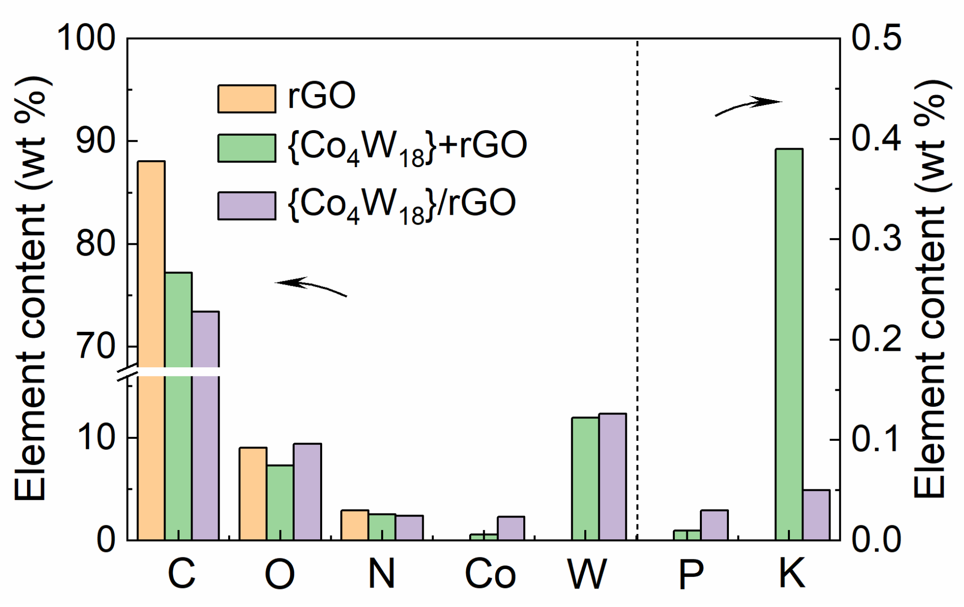

Supplementary Figure 2 Element analysis of materials by EDS, wt %.

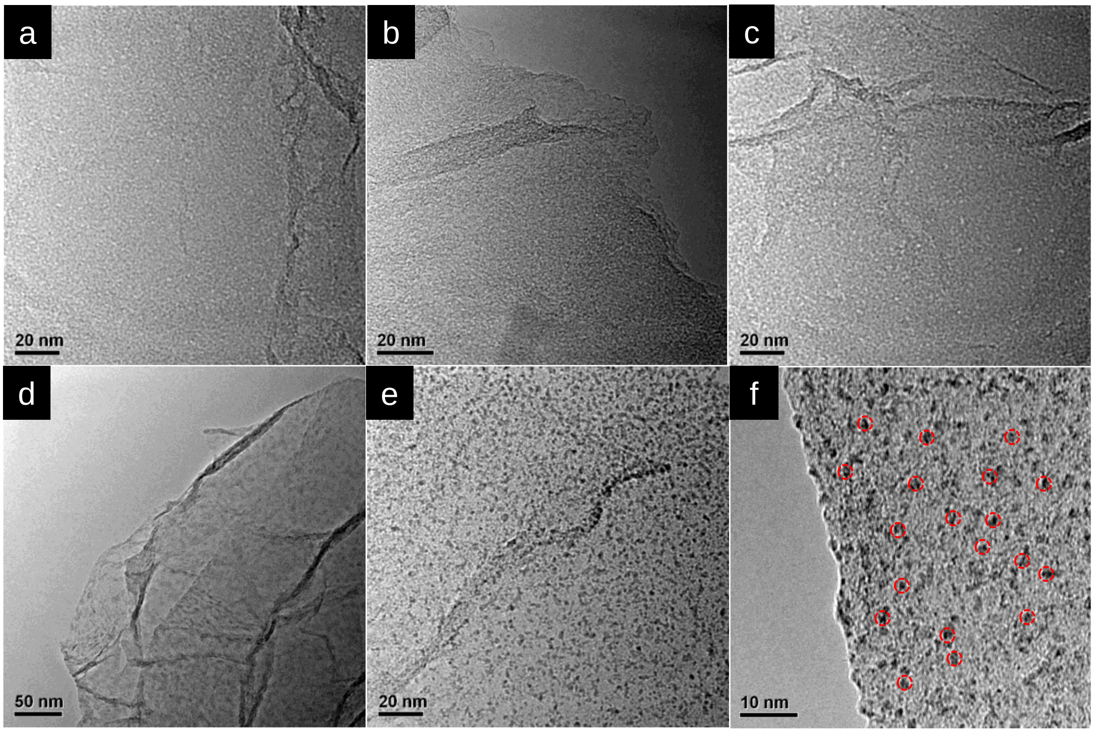

Supplementary Figure 3 TEM images of materials: (a-c) rGO, and (d-f) $\{Co_4W_{18}\}$/rGO.

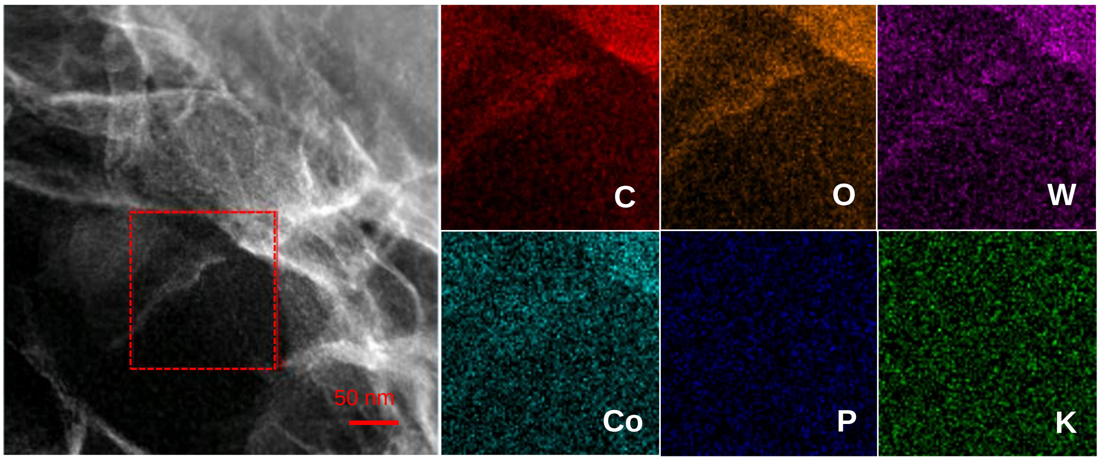

Supplementary Figure 4 Elemental mapping images of the {Co_4W_{18}}/rGO sample.

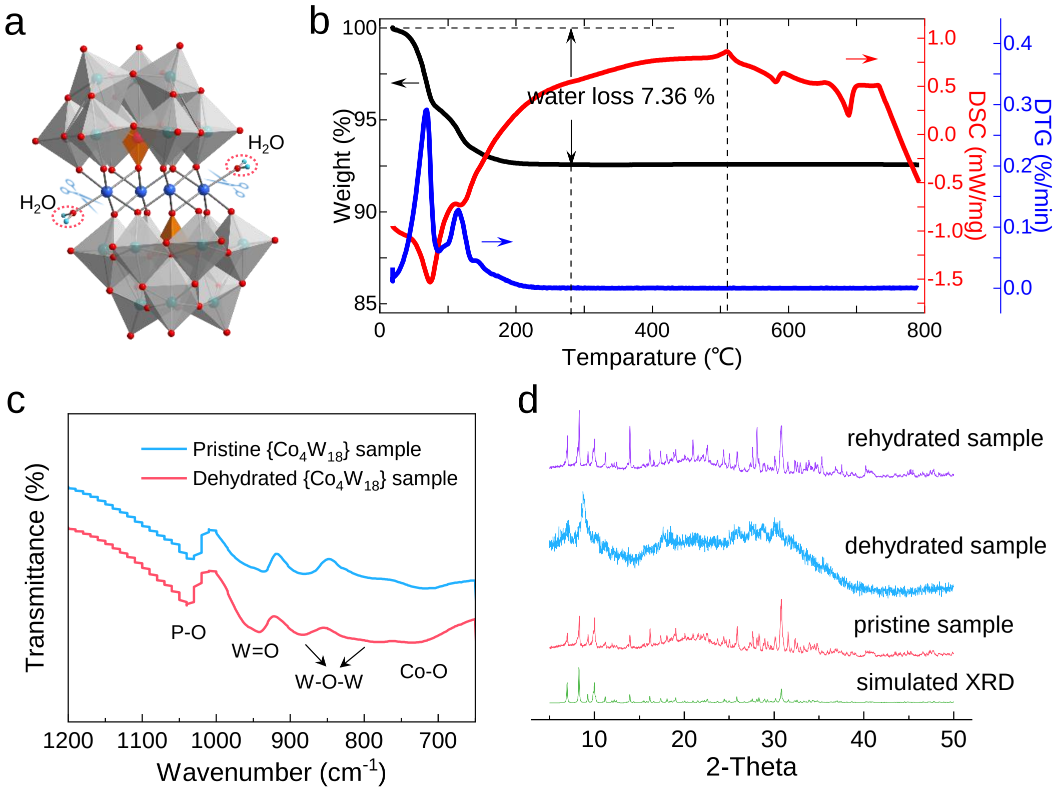

Supplementary Figure 5 The thermal stability of {Co_4W_{18}} clusters: (a) Schematic diagram of sandwich-like molecular structure. (b) Thermogravimetric analysis, (c) FT-IR spectra and (d) XRD patterns.

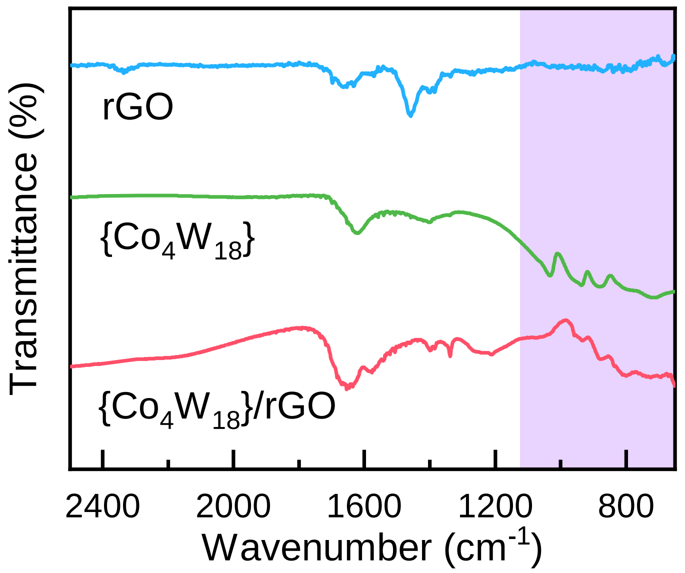

Supplementary Figure 6 The FT-IR spectra of rGO, $\{Co_4W_{18}\}$ and $\{Co_4W_{18}\}$/rGO.

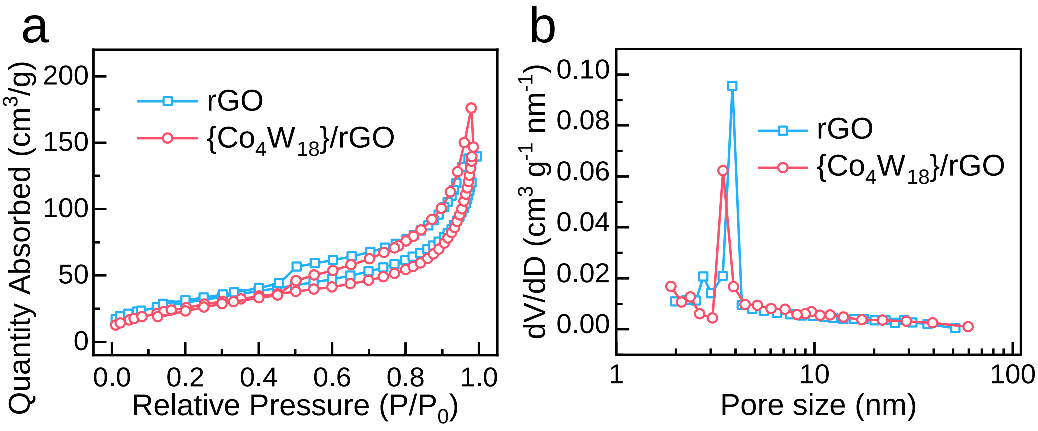

Supplementary Figure 7 BET tests of rGO and $\{Co_4W_{18}\}$/rGO samples: (a) N_2 adsorption-desorption isotherm loop. (b) pore-size distribution plot.

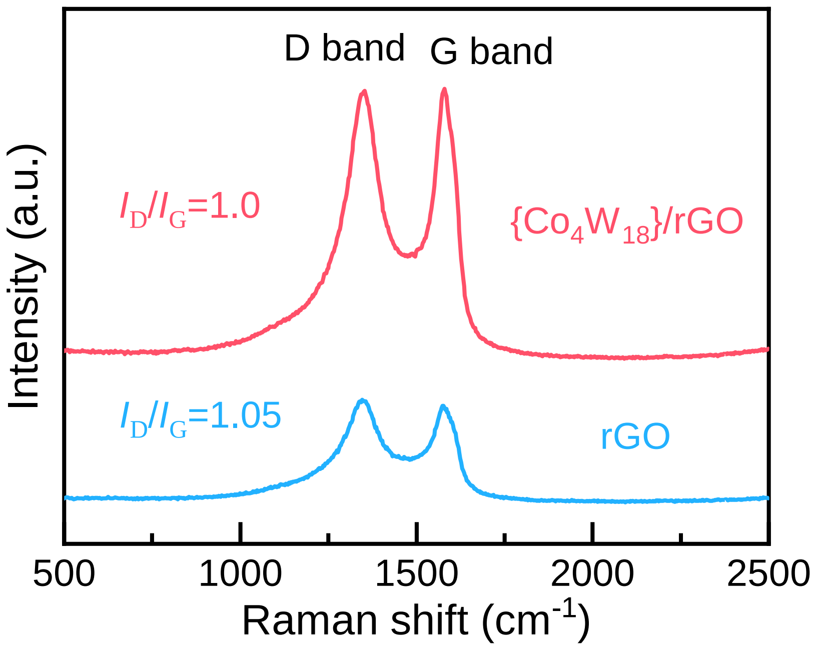

Supplementary Figure 8 Raman tests of rGO and $\{Co_4W_{18}\}$/rGO samples.

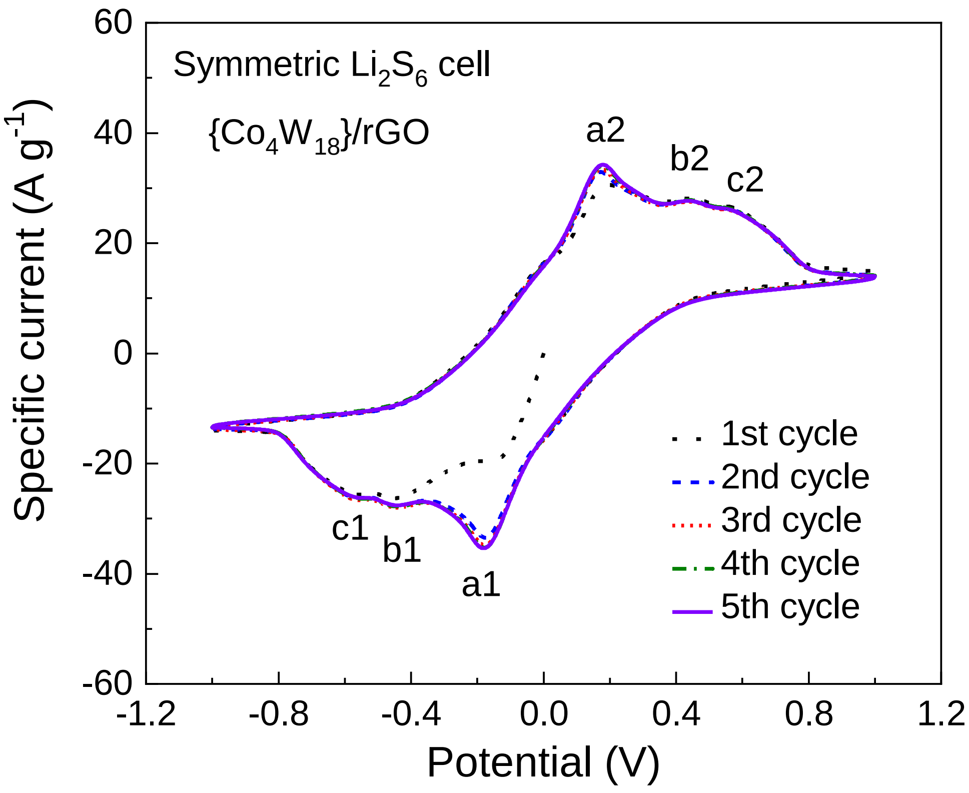

Supplementary Figure 9 CV curves of Li_2S_6 symmetric cells with $\{Co_4W_{18}\}$/rGO electrodes at the first five cycles.

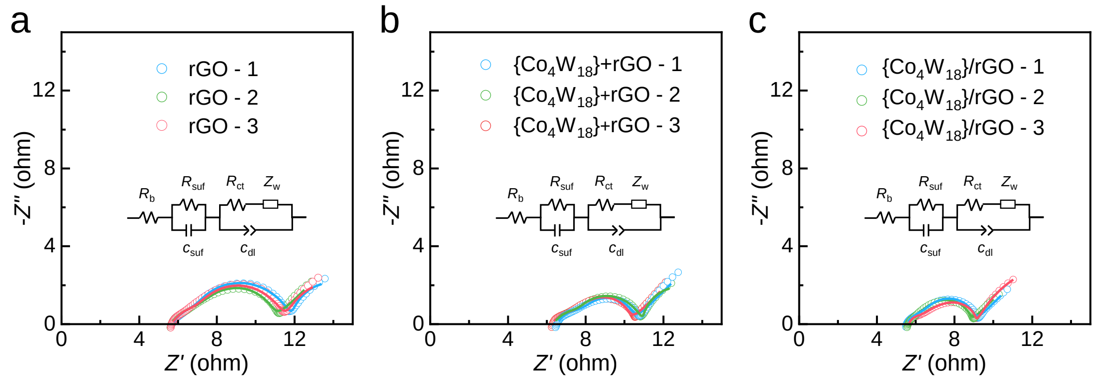

Supplementary Figure 10 The raw (symbols) and fitting (lines) impedance data of three identical Li_2S_6 symmetrical cells with the (a) rGO, (b) {Co_4W_{18}}+rGO and (c) {Co_4W_{18}}/rGO electrodes. The insets are the equivalent circuits.

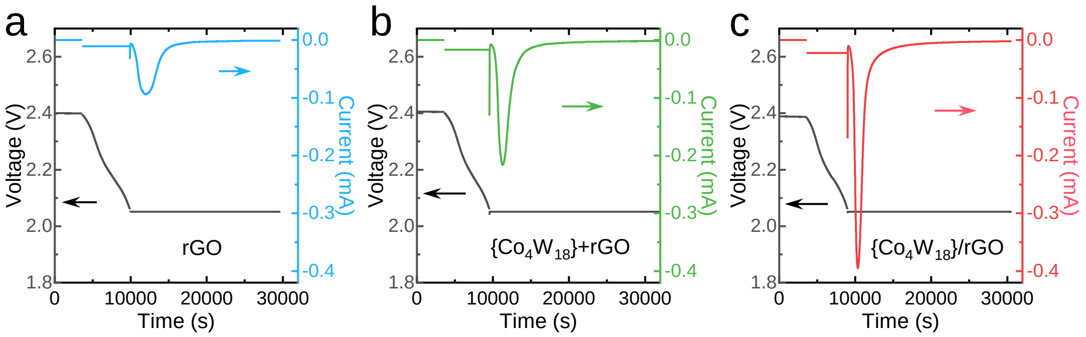

Supplementary Figure 11 The corresponding galvanostatic and potentiostatic curves of potentiostatic nucleation experiments on the rGO, $\{Co_4W_{18}\}$+rGO, $\{Co_4W_{18}\}$/rGO electrodes.

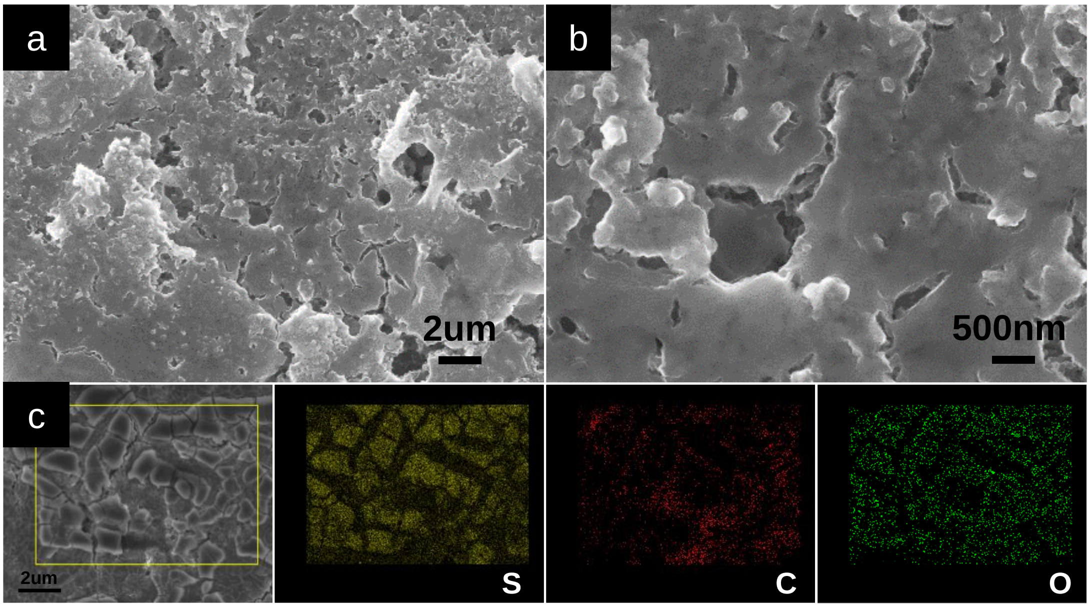

Supplementary Figure 12 (a-b) SEM images, (c) Elemental mapping images of rGO electrodes after potentiostatic nucleation.

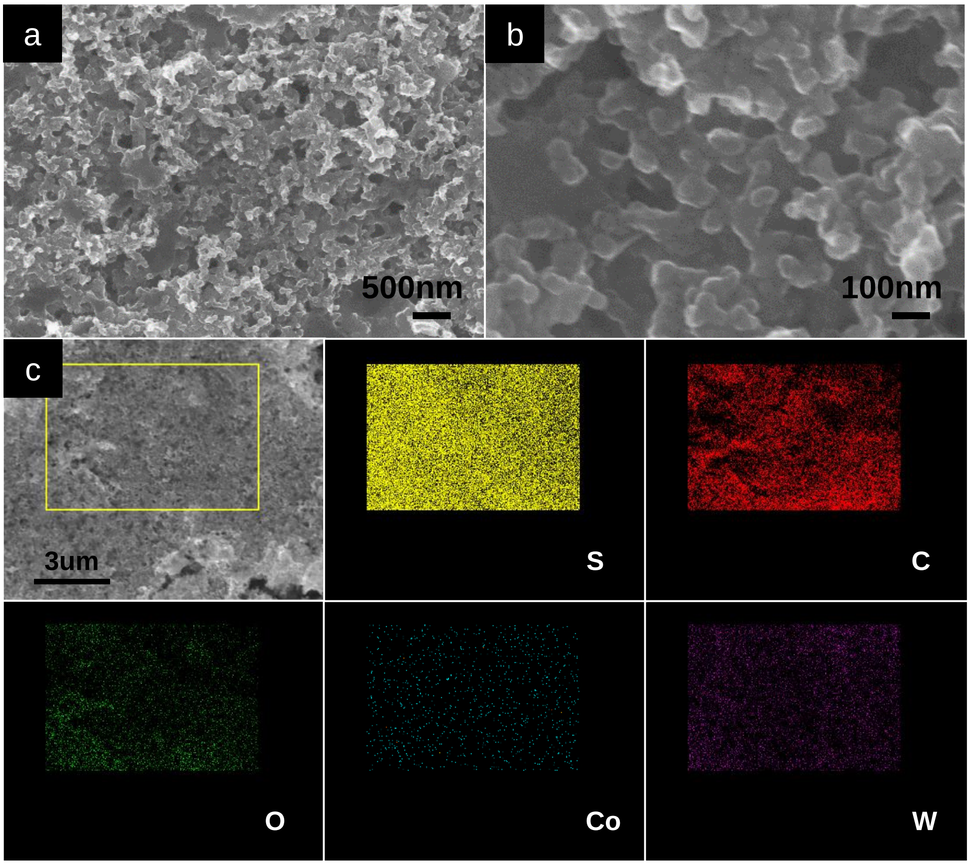

Supplementary Figure 13 (a-b) SEM images, (c) Elemental mapping images of $\{Co_4W_{18}\}$/rGO electrodes after potentiostatic nucleation.

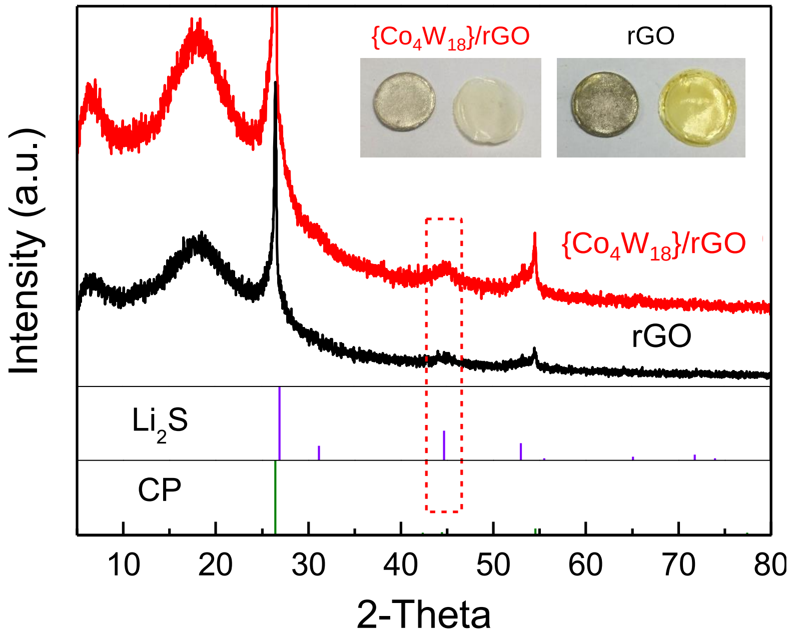

Supplementary Figure 14 XRD patterns of rGO and $\{Co_4W_{18}\}$/rGO electrodes after potentiostatic nucleation. The insets are the optical images of the lithium anodes and separators. CP is the abbreviation of the carbon paper collector.

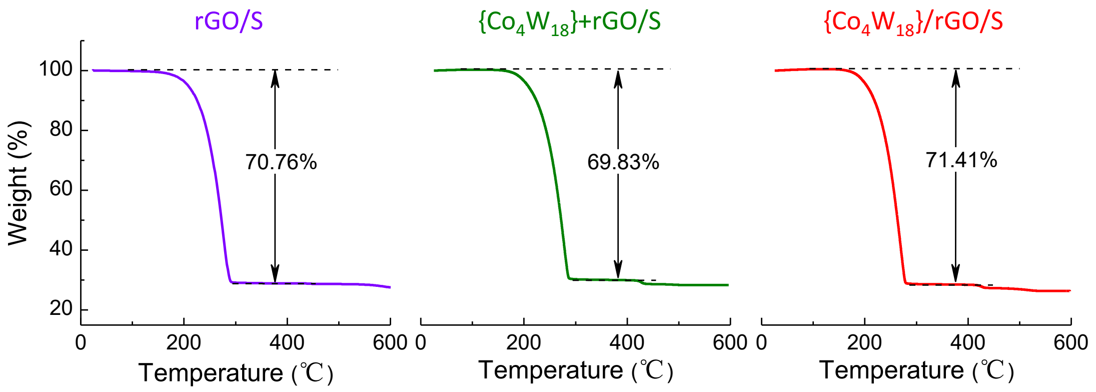

Supplementary Figure 15 Thermogravimetric analysis of rGO/S, $\{Co_4W_{18}\}$+rGO/S and $\{Co_4W_{18}\}$/rGO/S composite electrodes.

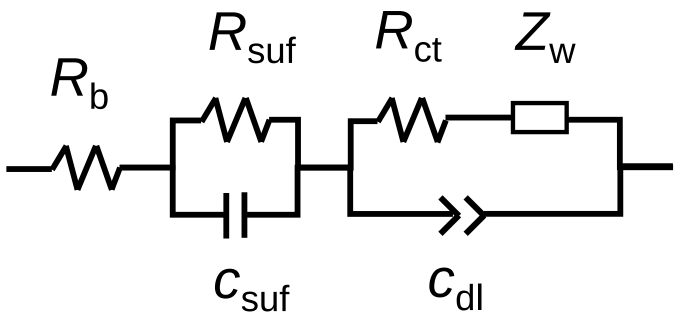

Supplementary Figure 16 The equivalent circuit of electrochemical impedance spectra of sulfur composite cathodes.

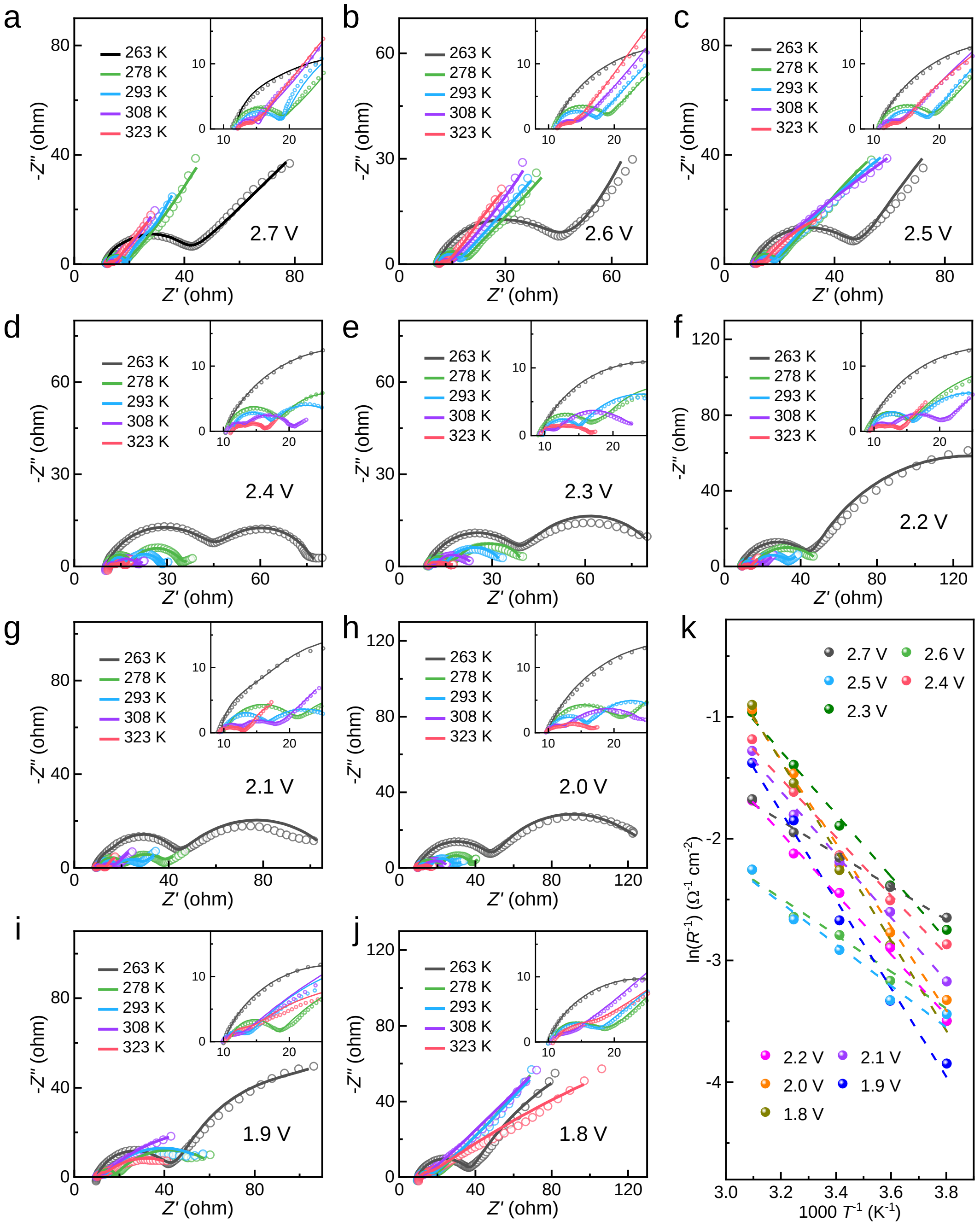

Supplementary Figure 17 The raw (symbols) and fitting (lines) impedance data of $\{Co_4W_{18}\}$/rGO/S cathodes from 2.7 V to 1.8 V and the corresponding Arrhenius fitting. The insets are the local magnification profiles.

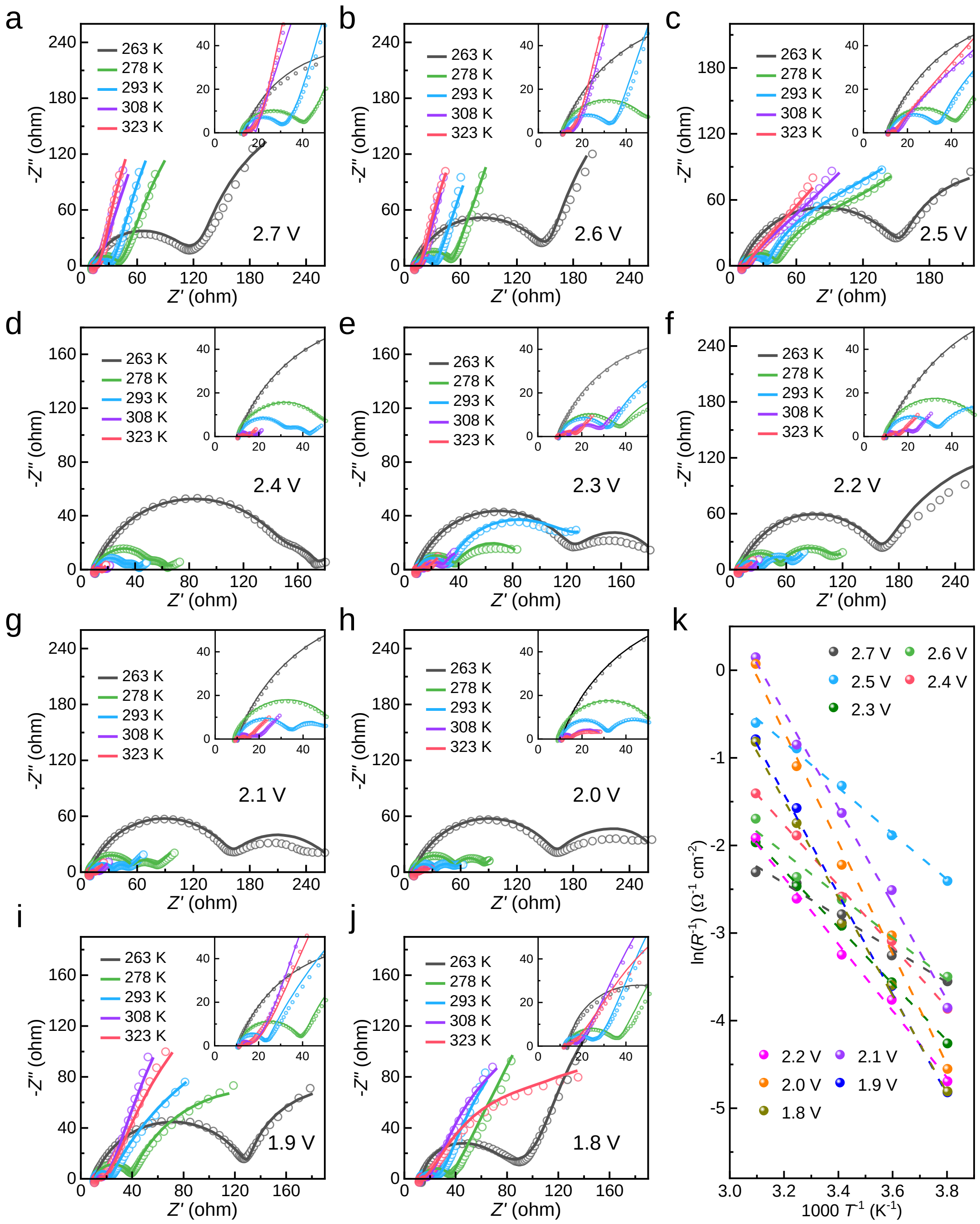

Supplementary Figure 18 The raw (symbols) and fitting (lines) impedance data of rGO/S cathodes from 2.7 V to 1.8 V and the corresponding Arrhenius fitting. The insets are the local magnification profiles.

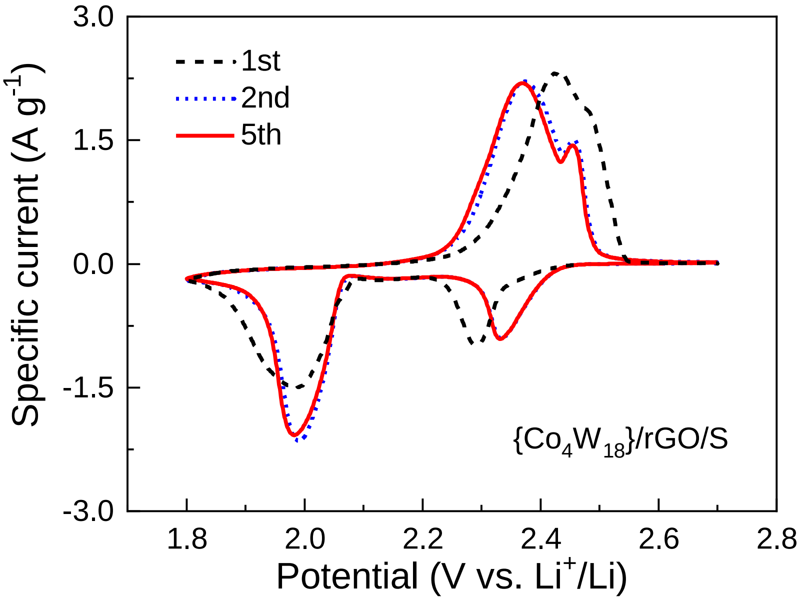

Supplementary Figure 19 CV curves of {Co_4W_{18}}/rGO/S cathode for five cycles at the scan rate of 0.1 mV/s.

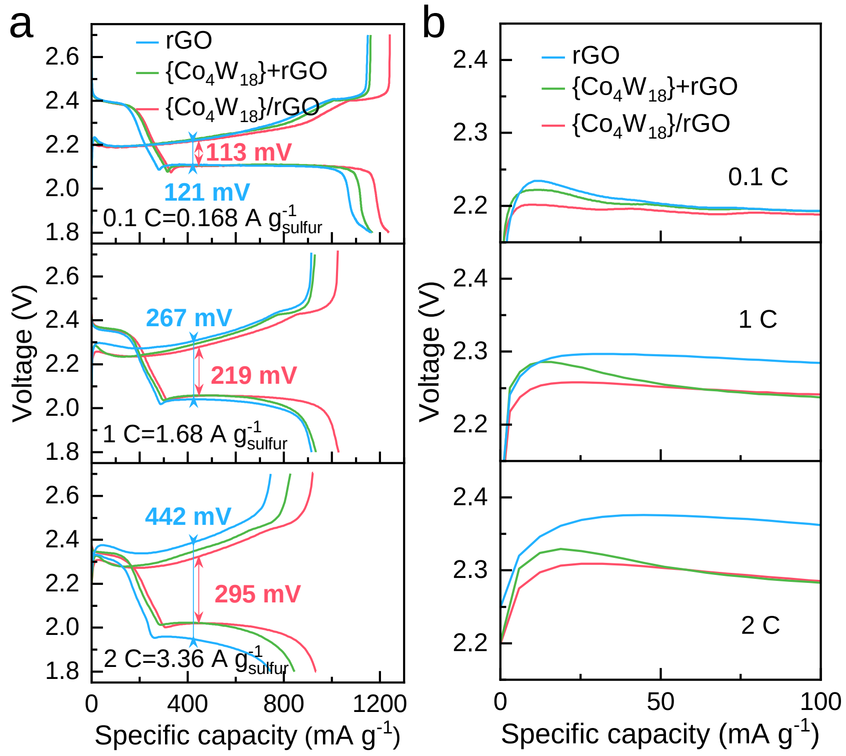

Supplementary Figure 20 (a) Galvanostatic discharge-charge curves of various sulfur cathodes at 0.1 C, 1 C and 2 C (i.e., 0.168, 1.68 and 3.36 A g_s^{-1}). (b) The activation barrier of Li_2S on rGO, $\{Co_4W_{18}\}$+rGO and $\{Co_4W_{18}\}$/rGO electrodes.

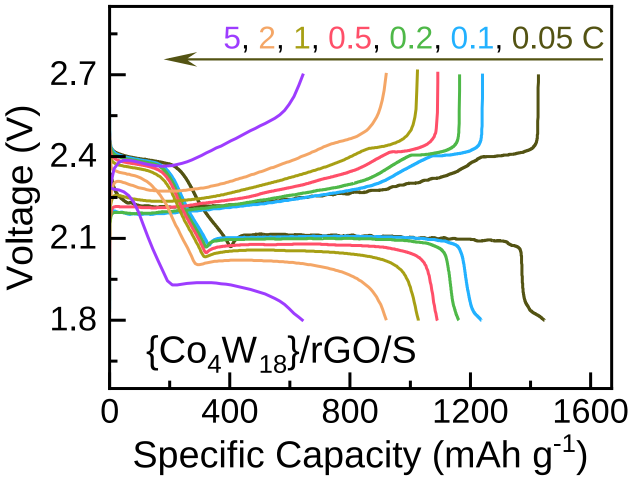

Supplementary Figure 21 Galvanostatic discharge-charge curves of $\{Co_4W_{18}\}$/rGO/S cathode at different rates (1 C corresponding the specific current of 1.68 A g_s^{-1}).

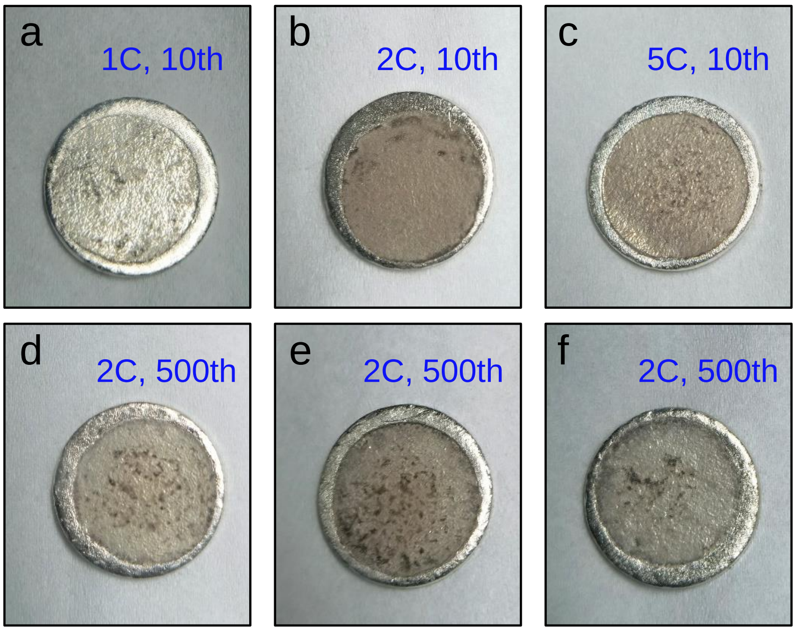

Supplementary Figure 22 The optical photographs of lithium metal electrodes after cycling at different rates (1C, 2C and 5C, i.e., 1.68, 3.36 and 8.4 A g_s^{-1}), which have been washed thoroughly with anhydrous DME solvent.

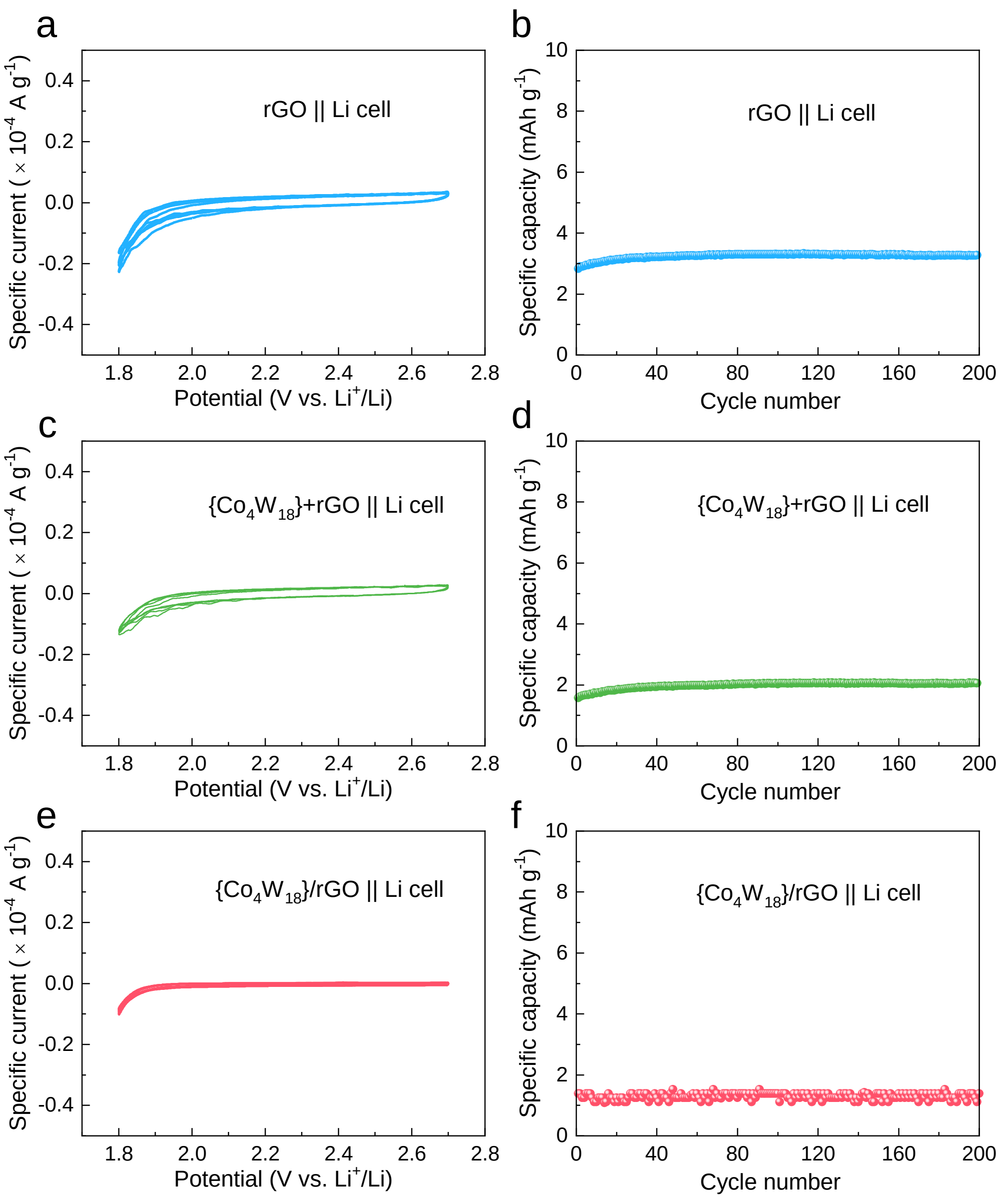

Supplementary Figure 23 CV curves of (a) rGO||Li, (c) $\{Co_4W_{18}\}$+rGO||Li and (e) $\{Co_4W_{18}\}$/rGO||Li cells at the scan rate of 0.1 mV s^{-1} in the voltage range of 1.8~2.7 V. And cycling performance of (b) rGO||Li, (d) $\{Co_4W_{18}\}$+rGO||Li and (f) $\{Co_4W_{18}\}$/rGO||Li cells at the current density of 50 mA g^{-1} in the voltage range of 1.8~2.7 V.

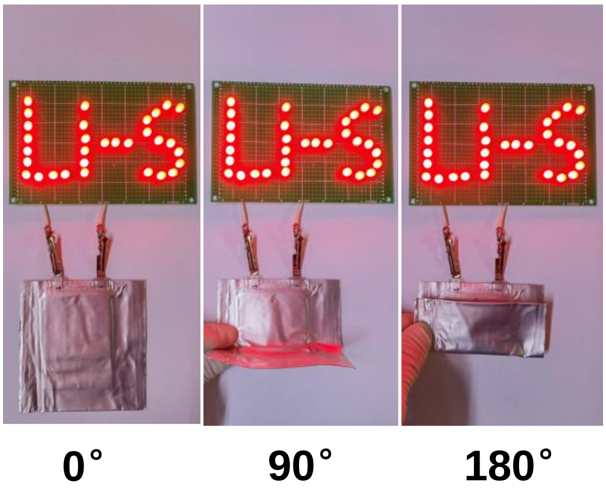

Supplementary Figure 24 Photographs of LED bulb circuit powered by pouch cells under various bending states.

Supplementary Table 1 Comparison of long-term cycle performance of this work with the state-of-the-art single-atom host materials for Li-S coin cells.

Materials	S loading (mg cm^{-2})	E/S ratio* (μL mg^{-1})	Capacity (mAh g^{-1})	Decay rate per cycle (%)	Ref.
{Co_4W_{18}}/rGO (S cathode)	1.5	16.7	670 (1000th cycle, 2 C) #	0.015	This work
Co-SAs in N-doped carbon nanosheets (S cathode)**	~1.2	31.85	675 (1000th cycle, 1 C)	0.035	1
Co–SAs in N-doped carbon matrix (S cathode)	2	13.5	737 (600th cycle, 1 C)	0.046	2
Co-Clusters in CNT/MoS_2 (S cathode)	1.5	12	486 (800th cycle, 1 C)	0.066	3
Mn-SAs in O-, N-doped hollow carbon sphere (S cathode)	~1.3	15	450 (1000th, 1 C)	0.05	4
Co-SAs in nanocarbon matrix (Li_2S cathode)	1.5~2.0 (Li_2S)	--	402 (based on S) (1500th cycle, 2 C)	0.04	5
Fe-SAs in N-doped porous carbon (Li_2S cathode)	2.0-2.3 (Li_2S)	--	490 (based on S) (1000th cycle, 2 C)	0.04	6
Fe-SAs in g-C_3N_4 (Li_2S_8 cathode)	2.3	~10	624 (1000th cycle, 2 C)	0.032	7

* E/S ratio = the ratio of the electrolyte dosage (μL) to total sulfur weight (mg), ** Co-SAs = Single Co atoms, # 1 C = 1.68 A g_s^{-1}, 2 C=3.36 A g_s^{-1}.

Note: the above electrochemical performance comparison is based on Li-S coin cells at room temperature using the flooded conventional ether-based electrolyte (DOL/DME, 1:1, by volume).

Supplementary Table 2 Electrochemical performance comparison with the state-of-the-art single-atom host materials based on high sulfur loading for Li-S coin cells.

Materials	S loading (mg cm^{-2})	E/S ratio (μL mg^{-1})	Areal capacity (mAh cm^{-2})	Ref.
$\{Co_4W_{18}\}$/rGO (S cathode)	5.6	4.5	4.55 (50th cycle, 0.1 C)	This work
Co-SAs in N-doped carbon nanosheets (S cathode)	4.9	10.4	4.24 (120th cycle, 0.2 C)	1
Co–SAs in N-doped carbon matrix (S cathode)	5	7.7	~2 # (150th cycle, 1 C)	2
Co-Clusters in CNT/MoS_2 (S cathode)	3.6	12	2.67 (100th cycle, 0.2 C)	3
Mn-SAs in O-, N-doped hollow carbon sphere (S cathode)	4	10	2.1 # (100th cycle, 1 C)	4
Co-SAs in N-doped carbon (S cathode)	4.3	5.7	4.02 # (100th cycle, 0.2 C)	8
Co-SAs in porous C_3N_4 nanosheets (polysulfide cathode)	4	18	3.12 # (200th cycle, 0.3 C)	9
V-SAs in N-doped graphene (S cathode)	5	--	2.42 # (200th cycle, 0.5 C)	10
Co-clusters in N-doped porous carbon (S cathode)	5.9	--	4.8 (100th cycle, 0.2 C)	11
Co-SAs in N-doped graphene (S cathode)	6	12	5.1 (100th cycle, 0.2 C)	12

the areal capacities are obtained from the specific capacities and sulfur loading in the corresponding references.

Note: the above electrochemical performance comparison is based on Li-S coin cells at room temperature using the conventional ether-based electrolyte (DOL/DME, 1:1, by volume).

Supplementary References

1 Li, Y. *et al*. Fast conversion and controlled deposition of lithium (poly)sulfides in lithium-sulfur batteries using high-loading cobalt single atoms. *Energy Storage Mater.* **30**, 250-259 (2020).

2 Li, Y. *et al*. Cobalt single atoms supported on N-doped carbon as an active and resilient sulfur host for lithium-sulfur batteries. *Energy Storage Mater.* **28**, 196-204 (2020).

3 Ma, Z. *et al*. Embedding Cobalt Atom Clusters in CNT-Wired MoS_2 Tube-in-Tube Nanostructures with Enhanced Sulfur Immobilization and Catalyzation for Li-S Batteries. *Small* **17**, 2102710 (2021).

4 Liu, Y. *et al*. O-, N-Coordinated single Mn atoms accelerating polysulfides transformation in lithium-sulfur batteries. *Energy Storage Mater.* **35**, 12-18 (2021).

5 Wang, J. *et al*. Single atomic cobalt catalyst significantly accelerates lithium ion diffusion in high mass loading Li_2S cathode. *Energy Storage Mater.* **28**, 375-382 (2020).

6 Wang, J. *et al*. Single-atom catalyst boosts electrochemical conversion reactions in batteries. *Energy Storage Mater.* **18**, 246-252 (2019).

7 Lu, C., Chen, Y., Yang, Y. & Chen, X. Single-atom catalytic materials for lean electrolyte ultrastable lithium-sulfur batteries. *Nano Lett.* **20**, 5522-5530 (2020).

8 Zhou, X. *et al*. Size-Dependent Cobalt Catalyst for Lithium Sulfur Batteries: From Single Atoms to Nanoclusters and Nanoparticles. *Small Methods* (2021), DOI: 10.1002/smtd.202100571.

9 Wu, J. *et al*. Cobalt atoms dispersed on hierarchical carbon nitride support as the cathode electrocatalyst for high-performance lithium-polysulfide batteries. *Sci. Bull.* **64**, 1875-1880 (2019).

10 Zhou, G. *et al*. Theoretical Calculation Guided Design of Single-Atom Catalysts toward Fast Kinetic and Long-Life Li-S Batteries. *Nano Lett.* **20**, 1252-1261 (2020).

11 Wang, R. *et al*. Highly Dispersed Cobalt Clusters in Nitrogen-Doped Porous Carbon Enable Multiple Effects for High-Performance Li-S Battery. *Adv. Energy Mater.* **10**, 1903550 (2020).

12 Du, Z. *et al*. Cobalt in Nitrogen-Doped Graphene as Single-Atom Catalyst for High-Sulfur Content Lithium-Sulfur Batteries. *J. Am. Chem. Soc.* **141**, 3977-3985 (2019).
